# Nanomaterials for Treating Bacterial Biofilms on Implantable Medical Devices

**DOI:** 10.3390/nano10112253

**Published:** 2020-11-13

**Authors:** Hoai My Tran, Hien Tran, Marsilea A. Booth, Kate E. Fox, Thi Hiep Nguyen, Nhiem Tran, Phong A. Tran

**Affiliations:** 1 Centre for Biomedical Technologies, Queensland University of Technology (QUT), 2 George Street, Brisbane, QLD 4000, Australia; h39.tran@hdr.qut.edu.au (H.M.T.); anhhien.tran@hdr.qut.edu.au (H.T.); 2 Interface Science and Materials Engineering Group, School of Mechanical, Medical and Process Engineering, Queensland University of Technology, Brisbane, QLD 4000, Australia; 3 School of Engineering, RMIT University, Melbourne, VIC 3001, Australia; marsilea.harrison@rmit.edu.au (M.A.B.); kate.fox@rmit.edu.au (K.E.F.); 4 Center for Additive Manufacturing, RMIT University, PO Box 2476, Melbourne, VIC 3001, Australia; 5 School of Biomedical Engineering, International University, Vietnam National University, Ho Chi Minh City 71300, Vietnam; nthiep@hcmiu.edu.vn; 6 School of Science, RMIT University, Melbourne, VIC 3001, Australia; nhiem.tran@rmit.edu.au

**Keywords:** bacteria, biofilm, nanomaterials, treatment, infection, in situ

## Abstract

Bacterial biofilms are involved in most device-associated infections and remain a challenge for modern medicine. One major approach to addressing this problem is to prevent the formation of biofilms using novel antimicrobial materials, device surface modification or local drug delivery; however, successful preventive measures are still extremely limited. The other approach is concerned with treating biofilms that have already formed on the devices; this approach is the focus of our manuscript. Treating biofilms associated with medical devices has unique challenges due to the biofilm’s extracellular polymer substance (EPS) and the biofilm bacteria’s resistance to most conventional antimicrobial agents. The treatment is further complicated by the fact that the treatment must be suitable for applying on devices surrounded by host tissue in many cases. Nanomaterials have been extensively investigated for preventing biofilm formation on medical devices, yet their applications in treating bacterial biofilm remains to be further investigated due to the fact that treating the biofilm bacteria and destroying the EPS are much more challenging than preventing adhesion of planktonic bacteria or inhibiting their surface colonization. In this highly focused review, we examined only studies that demonstrated successful EPS destruction and biofilm bacteria killing and provided in-depth description of the nanomaterials and the biofilm eradication efficacy, followed by discussion of key issues in this topic and suggestion for future development.

## 1. Introduction

**Figure 1:**
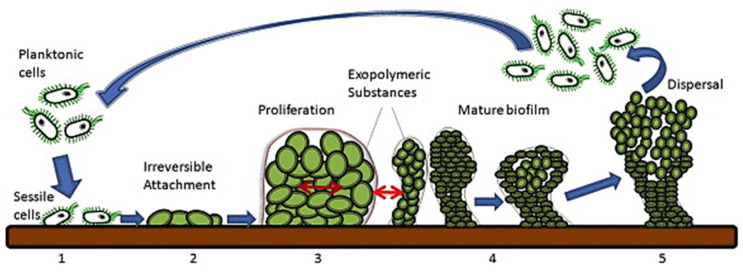
Biofilm formation and dispersion. (**1**) Bacterial cell attachment onto the surface. (**2**) Bacterial cells becoming irreversibly attached. (**3**) Bacterial proliferation and EPS secretion. (**4**) Biofilm formation and maturation. (**5**) Biofilm dispersal and mobility of planktonic cells (reproduced from [12] with permission from Elsevier, 2015).

Despite hygienic techniques and prophylactic antibiotics, infection rates of primary implants remain clinically significant. In the US alone, infections related to medical devices account for 45% of total nosocomial (hospital-based) infections [1], with approximately one million cases per year [2]. Device-associated infection is predominantly linked with bacterial biofilm formation. Biofilms can form on most medical devices from a wide range of bacterial species [3]. The process initiates with bacterial adherence to the device surfaces or the compromised surrounding tissue. Bacteria then proliferate, colonize and form the biofilm consisting of bacterial cells enclosed in the secreted extracellular polymeric substances (EPS) (Figure 1) [4,5]. The EPS comprises polysaccharides, nucleic acids, proteins and lipids [5,6] with an acidic microenvironment caused by hypoxia and anaerobic glycolysis [6,7,8]. The biofilm provides bacterial cells with protection against shear stress, host immune defence and antibiotic actions [9,10,11]. Moreover, bacterial cells released from biofilms and can cause local recurrence or infection at other sites by haematogenous dissemination [3,10].

One major strategy in the infection control of implantable medical devices is to prevent biofilm formation. The gold standard for biofilm prevention involves aseptic techniques and antibiotic prophylaxis [13,14]. Systemic administration and local antibiotic reservoirs have been frequently employed as prophylactic means in clinical settings [15,16,17,18]. More advanced preventive measures, still in research and development, include new biomaterials with intrinsic antimicrobial properties or surface modification with antimicrobial moieties. The main rationales behind these designs are based on the concept of ‘race for the surface’ put forward by Gristina in 1987 [19], which postulated that bacteria and mammalian cells compete for available binding sites on the implant surface and implant success depends largely on who wins this competition. Prevention of bacterial colonization and biofilm formation thus should allow tissue cells to form a natural protective layer on the implant. Yet, despite a large number of in vitro and in vivo studies demonstrating promising biofilm-preventing results [20,21,22], only very few have been successfully translated to clinical applications. For example, besides local antibiotic depots that were not specifically designed to act as antimicrobial implant surface treatments, only four technologies have become commercially available technologies or have shown clinical efficacy in orthopaedics and trauma for clinical use, including silver coating, iodine coating, gentamicin-poly(d,l-lactide) (PLLA) coating, and PLLA-linked hyaluronan hydrogel coating containing antibiotics [23]. Even so, the efficacy of these four technologies in large clinical trials remain to be proven.

Clinically, treating device-associated biofilms is most relevant in device/implant retention surgery. In contrast to the common one- or two-stage device exchange surgeries [24] where infected devices are removed and replaced by a new implant, the implant retention surgery (also known as ‘debridement, antibiotics and implant retention—DAIR’) is less invasive and preferable in scenarios such as early or acute infection. The success rate of DAIR heavily depends on the ability to effectively eradicate the biofilms [25], yet it is well recognized that the biofilm’s EPS provides a protective matrix which reduces the penetration of antimicrobial agents and the embedded, dormant biofilm bacteria are highly resistant to conventional antimicrobial agents such as antibiotics [26].

There are already many excellent reviews on medical device-associated bacterial biofilms, their implication in infections, or strategies to prevent their formation, such as those by Rodney [27], Hall-Stoodley [28], or Ferraris and colleagues [29,30]. Several recent publications have also reviewed the methods of eradicating these biofilms, such as the work by Verderosa et al. [31]. In the current paper, we focused on reviewing nanomaterials, a subset of biomaterials, in eradicating bacterial biofilm. In particular, we only examined studies that demonstrated success in destroying the EPS and killing the embedded bacteria rather than preventing biofilm formation. Here, we focused on the key, most promising nanomaterials and provided details of the most significant results to offer a highly focused and in-depth review of the topic and provide readers with clear comparison of the synthesis and effectiveness of these materials to guide future development.

## 2. Nanomaterials for Treating Bacterial Biofilms on Medical Devices

**Figure 2:**
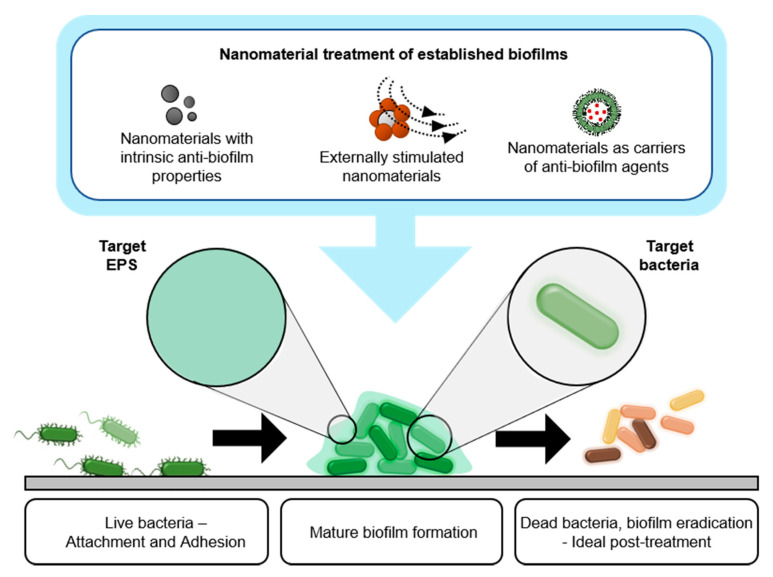
Schematic indicating the growth of mature bacterial biofilm and three major types of nanomaterials for biofilm treatment: (i) intrinsically biofilm-eradicating nanomaterials, (ii) nanocarriers of biofilm-eradicating agents, and (iii) responsive biofilm-eradicating nanomaterials.

In this section, we categorized the nanomaterials in biofilm treatment into three groups, including (i) nanomaterials with intrinsic biofilm-eradicating properties, (ii) nanomaterials as delivery vehicles for biofilm-eradicating agents, and (iii) responsive nanomaterials (Figure 2).

### 2.1. Nanomaterials with Intrinsic Biofilm-Eradicating Properties

Despite the fact that there is a large number of nanomaterials with intrinsic bactericidal properties, which have been reviewed elsewhere, only some nanomaterials have been shown to have the intrinsic abilities to destroy the EPS or killing bacterial biofilm [32,33]. Most of the intrinsic biofilm-eradicating nanomaterials are derived from bactericidal nanomaterials and thus they were often also demonstrated to have effects on biofilm formation as reviewed below.

A key type of nanoparticle with intrinsic anti-biofilm activities is metallic nanoparticles. Certain metallic nanoparticles can release soluble ions that target bacteria or the EPS; some can interact with the biofilm’s EPS as a result of surface functional groups or charge interaction [6,34,35,36,37]. Silver is among the most studied metallic nanomaterials with high bactericidal properties on a broad spectrum of microorganism species and has been shown to be able to eradicate biofilms. In one study, Salunke et al. [38] synthesized AgNPs from plant extract and investigated their antimicrobial properties on *Acinetobacter baumannii*, *E. coli* and *S. aureus*, using the disk diffusion test. They showed that AgNPs inhibited bacterial growth with low minimum inhibitory concentration (2 µg/disk against *E. coli* and 8 μg/disk against *A. baumannii* and *S. aureus*), compared to the chemically synthesized AgNPs used as a control. In biofilm disruption assays, the biofilms were grown for 24 h. The results show that AgNPs could disrupt 88%, 67%, and 78% of *A. baumannii*, *E. coli* and *S. aureus* biofilms, respectively; and 64% of mixed biofilm after 24 h treatment at 37 °C [38]. In another study, Gaidhani et al. used *Acinetobacter calcoaceticus* to synthesize silver nanoparticles (AgNPs) with sizes ranging from 4–40 nm and showed that the nanoparticles were able to disrupt biofilms of 20 different pathogenic bacteria [39].

Some researchers showed anti-biofilm activity of gold nanoparticles. For example, Habimana et al. [40] developed gold particles (GNP) functionalized with enzyme proteinase K (denoted as GNP + PK) and tested them against *Pseudomonas fluorescens* biofilms which were formed for 72 h. The biofilm volume, measured from 3D confocal laser scanning microscopy, was found to decrease by 40%, 77%, and 74% after 24 h treatment with proteinase K (PK), GNP, and GNP + PK, respectively. The combined GNP + PK was able to reduce the biofilm thickness by 78%, while PK and GNP induced a reduction of 52% and 72%, respectively. Overall, the GNP + PK particles displayed greater antimicrobial/antibiofilm effects than either PK or GNP alone by causing structural damage to the biofilm, killing sessile cells within the biofilm and mechanically dispersing the cells in suspension [40].

**Figure 3:**
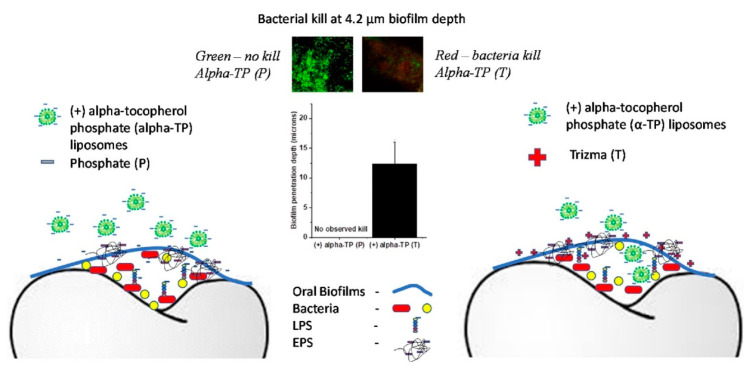
Schematic of biofilm elimination by combination of charge screening and alpha-tocopherol phosphate (α-TP) charged liposome nanoparticles. The penetration of negatively charged α-TP liposomes were enhanced in the Tris buffer (positively charged) causing bacteria death as compared to poor penetration and ineffective in phosphate buffer saline (PBS) (reproduced from [47] with permission from Elsevier, 2015).

Several research groups have also investigated polymeric nanomaterials with inherent anti-biofilm properties. These materials often work through mechanisms related to electrostatic interactions with bacteria and biofilm’s EPS, which usually have a negatively charge on the outer layer [41,42,43] Chitosan is a commonly used charged polymer that has high antibacterial activity. Mohamed et al. developed chitosan nanoparticles and tested on multi-species biofilm in the root canal, which inhibited 97% and 94% of the formation of biofilm in single and mixed-species biofilm, respectively. Furthermore, the chitosan nanoparticles also gradually disrupted the one-week pre-formed mixed-species biofilm for 8 days (5-log reduction) [44]. In another study, chitosan nanoparticles were tested against a 7 day old *E. faecalis* biofilm. After incubating with chitosan nanoparticles at 37 °C for 72 h, the authors found that the chitosan concentration of 5 mg/mL induced a maximum reduction in biofilm bacteria viability of 4 logs; and at the concentration of 20 mg/mL, most bacterial cells were completely killed [45]. In another study, Zhu et al. [46] showed that cationic PLGA nano-polymer could inhibit the growth of *Streptococcus mutants* over a 24 h period, with most bacteria being killed after 90 min. Furthermore, the cationic nano-polymer totally disintegrated 1 day old biofilms and induced 73% bacterial death at the concentration of 100 µg/ML [46]. In yet another study, Harper et al. [47] combined the effects of electrolyte charge screening and anionic (+) alpha-tocopherol phosphate (α-TP) liposome nanoparticles to enhance the diffusion through biofilm of the latter (Figure 3). The bacterial biofilm was grown for 18 h before being treated with the nanoparticles for 2 min. The self-assembled 700 nm liposomes had a zeta potential of −20 mV and could not penetrate the oral multispecies oral biofilms (from a donor) in a phosphate (-ve) buffer. In a tris(hydroxymethyl)aminomethane (+ve) buffer, the liposomes were found to penetrate 12.4  ±  3.6 μm into the biofilms and kill the embedded microbes [47].

### 2.2. Nanomaterials as Carriers of Biofilm-Eradicating Agents

The interactions between the biofilm, bacteria and antimicrobial agents are crucial in treating bacterial biofilms [6]. For therapies targeting biofilm bacteria, penetration into the biofilm is crucial. Antimicrobial compounds may not be able to reach the bacteria in a mature biofilm for several reasons, such as reduced diffusion across the EPS, drug degradation, or the poor interactions with biofilm components [48,49,50]. Nanoparticles can act as drug delivery vehicles that could address these problems. These nanocarriers with their intrinsic high surface-area-to-volume ratio and rich surface chemistry can be designed to have a controllable therapeutic dose, improve the stability of their ‘cargo’, or temporal and spatial cargo release. Nanocarriers can be made from various organic (e.g., lipid and polymer) and inorganic materials [49,51] and some even have intrinsic antimicrobial or antibiofilm properties [52,53].

A straightforward design involves using antibiotics in nanocarriers. In one example, Elbi et al. [54] developed chitosan nanoparticles containing ciprofloxacin and coated with Fucoidan to enhance antimicrobial activity and the endocytosis of the material into macrophages to kill intracellular bacteria. The authors showed that the nanocarriers effectively dispersed the 2 day old *S. Paratyphi A* biofilm, significantly more effective than ciprofloxacin alone (1.6 times better) and were not toxic to RAW 264.7 macrophages after 48 h of treatment. Furthermore, the nanocarriers also entered the macrophages and killed the intracellular *S. Paratyphi A* in a coculture model [54]. Mu et al. [55] utilized a similar concept for *P. aeruginosa* and *S. aureus* biofilm treatment. In this case, gentamicin was loaded in phosphatidylcholine-decorated AuNPs (GPA NPs) and the loaded nanoparticles were shown to eliminate 78%, 65%, 71% and 69% of the biofilms of *P. aeruginosa*, *S. aureus*, *E. coli*, and *L. monocytogenes*, respectively. Furthermore, GPA NPs were nontoxic to murine macrophage cells and also reduced the viability of intracellular bacteria from 5 × 10^7^ to 1 × 10^5^ CFU and 3.5 × 10^6^ to 0.5 × 10^6^ CFU for *P. aeruginosa* and *L. monocytogenes* in the infected macrophages, respectively [55].

Besides nanocarriers containing antibiotics, other nanocarrier systems have also been developed to deliver non-drug agents, such as enzymes or essential oils. Tan et al. [53] utilized positively charged chitosan nanoparticles as a nanocarrier for deoxyribonuclease I (DNase I, used to target biofilm’s extracellular DNA) and oxacillin to target *S. aureus* biofilms. The loaded polymer nanoparticles were shown to eradicate 70% and 100% of a *S. aureus* mature biofilm at oxacillin concentration of 0.0625 and 2 µg/mL, respectively after 24 h of treatment [53]. In another example, poly(lactic-co-glycolic acid) (PLGA) nanoparticles coated with poly(lysin) (PL) were used as the nanocarriers for ciprofloxacin (Cip) and DNase I. Tested nanoparticle formulations comprised PLGA-PL-Cip, PLGA-PL-Cip + PLGA-PL-DNase I, and PLGA-PL-Cip-DNase I. The nano-systems were investigated against a biofilm of the Gram-negative bacteria *P. aeruginosa*. In this study, the biofilm was cultivated at 37 °C in flow cell chamber for 4 days and treated with different nano-formulations for 24 h and the biomass and thickness of the biofilm were evaluated. The biomass and thickness reduced by 24% and 50%, respectively, in the PLGA-PL-Cip group, and by 29% and 52% in the combined PLGA-PL-Cip + PLGA-PL-DNase I group. In particular, the last group (PLGA-PL-Cip-DNase I) showed the best efficacy with a reduction of 43% in biomass and 63% in thickness. Moreover, this formulation also eradicated over 95% of the 2 day old biofilm at a concentration of antibiotic as low as 0.0078 µg/mL [56].

**Figure 4:**
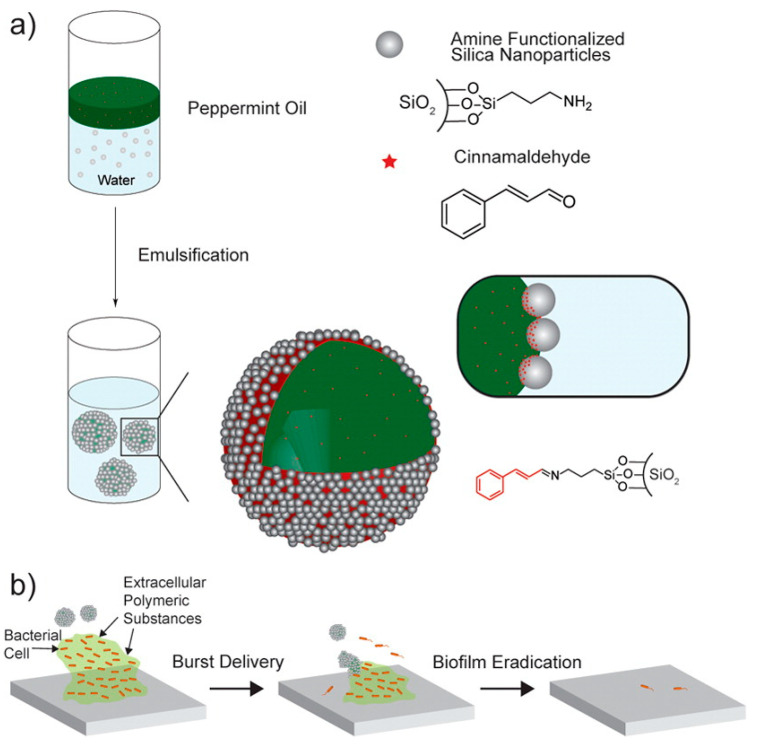
(**a**) Schematic describing the preparation of nanocarriers loaded with antimicrobial agents by an emulsification method, and (**b**) illustration of functionalized silica nanocarriers for bacterial biofilm treatment (reproduced from [57] with permission from American Chemical Society, 2015).

Duncan et al. [57] evaluated the use of silica nanoparticles functionalized with either peppermint oil (P-Cap) alone or in combination with cinnamaldehyde (CP-Cap) to disrupt and destroy *E. coli DH5*, *P. aeruginosa*, *S. aureus*, and *Enterobacter cloacae* complex biofilms (Figure 4). The biofilm was cultivated for 1 day before being treated with nanoparticles. After 3 h of CP-Cap treatment, the results show that these nanoparticles had the ability to reduce of the biofilm bacteria viability by 100% in all biofilms of *P. aeruginosa*, *E. coli*, *S. aureus*, and *E. cloacae* complex at 2% of emulsion solution. Meanwhile, bacterial colony forming units of *E. coli* biofilm also decreased by 4 logs after 3 h treatment of CP-Cap in a co-culture experiment. This nanoparticle formulation displayed the highest efficacy of biofilm eradication and bacterial killing among the formulations tested. The P-Cap and control groups, in contrast, were not effective [57].

Landis et al. [58] investigated the potential of using oil-in-water emulsification to fabricate nanocomposites containing carvacrol oil (X-NCs) to treat *P. aeruginosa*, *S. aureus*, *E. coli*, and *E. cloacae* biofilms. After 3 h of treatment, the bacterial viability in 1 day old biofilms was remarkably reduced by 95–90% for all bacterial types. Landis also investigated the effectiveness of X-NCs in a coculture by seeding the bacterial cells with a monolayer of fibroblasts overnight to form the biofilm. After 3 h of treatment with 10 wt% X-NCs, the number of biofilm bacteria was reduced 4 log, whilst the viability of the fibroblasts was maintained [58].

Another group of nanocarriers are based on the delivery of nitric oxide (NO) as a reactive free radical. Several studies have used this agent in the form of nanoparticles for biofilm eradication. As an example, Slomberg et al. [59] conjugated NO to silica nanoparticles and tested them against *S. aureus* and *P. aeruginosa* biofilms. The biofilms were cultivated for 48 h in a bioreactor before being treated with the nanoparticles for 24 h. The results show that the size and shape of the nanoparticles influenced the efficacy of biofilm eradication. The smaller size of silica particles displayed higher ability in treating *P. aeruginosa* biofilm, with the minimum bactericidal concentration (MBC) of 6 mg/mL for the 14 nm particles compared with 10 mg/mL for the 150 nm particles. Moreover, the spherical shape of silica particles showed MBC of 12 and 8 mg/mL for *S. aureus* and *P. aeruginosa*, respectively, compared with 4 and 1 mg/mL for rod shape particles [59]. Mihu et al. synthesized NO-containing nanoparticles from a combination of polyethylene glycol, glucose, chitosan and sodium nitrite. It was found that the nano-platform was able to interfere with biofilm formation on central venous catheters both in vitro and in vivo. In an in vitro biofilm formation study, a 5 mm-long catheter was placed into the MRSA culture and incubated for 24 h at 37 °C under shaking condition to form the biofilm. The colonized catheter was then treated with 5 mg/mL of NO-nanoparticles for 24 h. The treatment resulted in a 75% decrease in biofilm bacteria viability compared to that of the untreated control. The authors also inserted catheters into veins of rats and inoculated the lumens with MRSA to form biofilm in vivo. After 24 h, a single dose of 5 mg/mL NO-nanoparticles or PBS (as a control) was injected into the catheter lumens to treat the biofilms. After 48 h, the authors found that NO-nanoparticle treatment resulted in roughly a 1 log reduction of CFU/cm^2^ compared to the control and that samples treated with NO-nanoparticles had only a small number of bacterial clusters without biofilm slime, compared to presence of abundant biofilm on the untreated control [60]. This study thus showed that these promising nanoparticles need to be further investigated and optimization.

### 2.3. Treating Biofilms with Responsive Nanomaterials

Magnetic field, light, or the pH of the micro-environment have been exploited to activate a nanomaterial or transform it into a more active species for destroying bacterial biofilms. These nanomaterials are often metallic nanoparticles or nanocarriers that exert physical disruption to bacterial biofilms [1,61]. Their advantages include rich surface chemistry and the nanoscale dimensions, which promote infiltration into the biofilms as well as targeting bacteria when activated or transformed. Additionally, some non-specific biofilm disruption mechanisms such as heat and physical force allow for a wide range of bacterial biofilms to be targeted [51,62].

#### 2.3.1. Magnetically Responsive Nanomaterials

The main mechanism of this strategy is that a magnetic field is used to exert forces on magnetic nanoparticles and thus facilitates the penetration of nanoparticles into the biofilm [63]. Taylor et al. fabricated iron oxide nanoparticles (IONs) conjugated with metal ions and showed that after 24 h treatment under the magnetic field, the magnetic IONs conjugated with zinc, silver, and iron ions at concentration 370 µg/mL could disrupt up to 85%, 40%, and 50% of the 24 h old *S. aureus* biofilm, respectively [64]. This concept was also applied by Leuba et al. [65] for the disruption of *S. aureus* biofilms where amine, carboxylate and isocyanate-functionalized superparamagnetic-ION were used to increase their diffusion into the biofilm and consequently enhance biofilm disruption. Amine, carboxylate, and isocyanate functionalized disrupted 28.1%, 33.5%, and 31.1% of 24 h old *S. aureus* biofilm after 24 h of treatment, respectively, higher than superparamagnetic IONs alone. This phenomenon was explained by the catabolism of carbon and nicotinamide adenine dinucleotide (NADH) generation involved in biofilm eradication [65]. A different study by Zhang et al. developed a nanohybrid complex containing silver and iron oxide. In the absence of a magnetic field, the nanohybrid complexes disrupted only 50% and 40% of *E. coli* and *P. aeruginosa* biofilms, respectively. However, the treatment efficacy was significantly improved (88% in *E. coli* and 90% in *P. aeruginosa*) in the presence of a magnetic field [66]. The study also showed that iron oxide promoted the penetration of silver nanoparticles, which then killed the microorganisms. This strategy is still at the proof-of-principle stage where the experiments are usually set up so that the nanoparticles are pulled toward biofilms grown on a substrate by using magnets placed underneath the substrate. In future clinical application, this method needs to be further optimized considering important factors such as the types of implants and their geometry/physiological locations.

#### 2.3.2. Local pH or Exogenous H_2_O_2_—Responsive Nanomaterials

**Figure 5:**
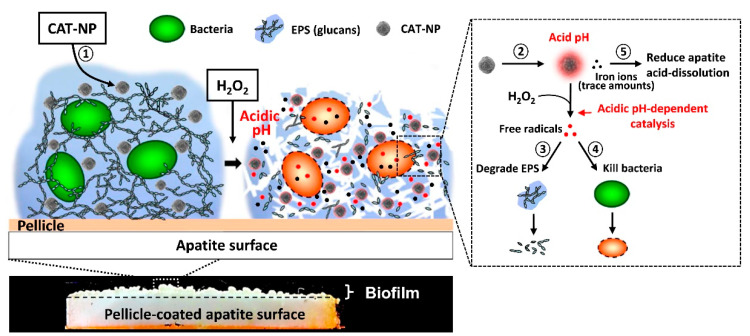
Schematic of biofilm disruption using hydrogen peroxide and iron oxide nanoparticles (ION). (**1**) The CAT-NP distributed in 3D biofilm structure after a brief topical exposure. (**2**) The CAT-NP catalyzed H_2_O_2_ via the Fenton reaction under acidic condition to produce free radicals. The generated free radicals (**3**) degraded EPS and (**4**) killed bacteria inside the biofilm. (**5**) CAT-NP were also able to release iron ions that can reduce acid dissolution of hydroxyapatite (reproduced from [70] with permission from Elsevier, 2016).

In an acidic environment, certain nanomaterials can catalyze the production of oxidative species. As a result, stimuli such as local pH and/or H_2_O_2_ can be exploited to assist in the treatment of biofilms. Gao et al. [67] developed iron oxide nanoparticles (ION) with peroxide-like activity for attacking bacteria (*E. coli*) and biofilm (*P. aeruginosa*). The nanoparticles catalyzed H_2_O_2_ into free radicals through the Fenton reaction [68,69]. This study showed that 82% of *P. aeruginosa* biofilm was eliminated and 98% of *E. coli* were killed within 5 min of exposure to peroxide-like ION [67]. A later study by Gao et al. also reported that peroxide-like ION could disrupt *S. mutants* oral biofilm in vitro and in vivo in rodent dental caries [70]. The particles were shown to kill 99.9% of *S. mutants* within 5 min and degraded approximately 57% of in vitro *S. mutants* biofilm. In vivo, the particles alone could reduce the severity of moderate and extensive stage caries in rodents after topical treatment. Meanwhile, the particles in combination with H_2_O_2_ were able to completely remove the extensive caries preventing the distinct cavitation. The particles were also non-toxic to other cell types and organs when being applied topically on rodent caries. The particles also released iron ions, which helped in decreasing the acid dissolution of bone mineral, hydroxyapatite. A schematic describing the effects of the nanoparticle platform on bacterial biofilms is shown in Figure 5, where the ION infiltrated the bacterial biofilm, and upon addition of H_2_O_2_ caused bacterial cell death and biofilm disruption. This peroxide-like ION was also found to be more effective than chlorhexidine, a commonly used antimicrobial agent [70].

A study by Horev et al. [8] provided an example of pH-activated nanomaterials. Here polymeric-based nanoparticles comprised of 2-(dimethylamino)ethyl methacrylate (DMAEMA), butyl methacrylate (BMA) and 2-propylacrylic acid (PAA) (p(DMAEMA)-b-p(DMAEMA-co-BMA-co-PAA)) containing farnesol for *S. mutants* biofilms treatment were developed. The nanoparticles showed high binding affinity to the biofilm and were also sensitive to an acidic environment, causing the release of free farnesol, a hydrophobic bactericidal agent. The results show that the nanoparticles effectively disrupted the biofilm and reduced 80% of bacteria and increased biofilm removal by two-fold after five applications over a 44 h period [8].

#### 2.3.3. Light/Heat—Responsive Nanomaterials

**Figure 6:**
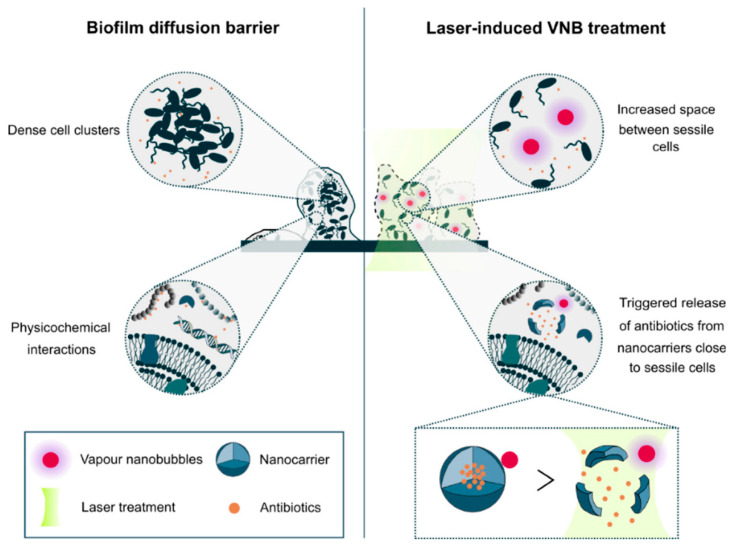
Laser-induced vapor nanobubbles (VNB) improved antibiotic delivery to biofilms. Impaired biofilm diffusion is caused by the fact that sessile cells cluster together into dense aggregates of hundreds of micrometers in size and because of the multi-component nature of the biofilm matrix which can trap molecules in their passage through biofilms. The mechanical impact of laser-induced VNB could increase the space between sessile cells, leading to a better flux and the effectivity of antimicrobial agents and their mechanical force can trigger antibiotic release from nanocarriers close to sessile bacteria (reproduced from [72] with permission from MDPI, 2019).

Another key strategy is photodynamic treatment or photothermal ablation, which is based on light-induced transformation or degradation of nanomaterials into biofilm-destroying species, or the generation of heat. Tierlinck et al. [71] investigated vapor nanobubbles (VNB) induced by the laser. The gold nanoparticles (70 nm in size) were able to deeply penetrate into the biofilm because of their small size. The gold nanoparticles (AuNPs) then received a high-energy laser pulse and induced rapid water evaporation and produced nanobubbles. The produced nanobubbles then created gaps within the biofilm, thus facilitating antibiotic penetration and transport. The results show synergy of VNB and tobramycin which resulted in approximately 80, 20, and 25 times CFU reduction in 24 h old *B. multivorans*, *P. aeruginosa*, and *S. aureus* biofilms after 3 min laser application and 24 h tobramycin treatment, respectively [71]. In a later study, the authors used these laser-induced VNB to functionalize the nanocarriers containing antibiotics for *P. aeruginosa* biofilm treatment (Figure 6). The AuNPs functionalized liposomes containing tobramycin reduced the CFU and biofilm biomass by approximately four times and 1.5 times as compared to untreated and liposomes containing tobramycin only, respectively [72].

Recently, graphitic carbon nitride (g-C_3_N_4_) has been shown as an efficient photocatalyst and photodegrading agent, which generated reactive oxygen species (ROS). Bing et al. [73] developed a nanoplatform of g-C_3_N_4_ nanosheets embedded with silver nanoparticles (Ag/g-C_3_N_4_). They showed that Ag/g-C_3_N_4_ nanoplatforms could effectively kill approximately 100% of *E. coli* and *S. aureus* at a very low concentration (50 µg/mL) after 30 min of activation by visible light. The nanoplatform at 200 µg/mL also cleaved the biofilm polysaccharide linkages and caused approximately 70% disruption of 48 h old *S. aureus* biofilm and killed the residue bacteria in the remaining biofilm after 3 h of activation by light [73]. Shrestha et al. [52] reported another approach for bacterial biofilm treatment using chitosan nanoparticles functionalized with Rose Bengal (RB), a photosensitizer agent, to enhance photodynamic therapy. The nanohybrids at concentration 0.1 mg/mL effectively killed 100% *Enterococcus faecalis* (*E. faecalis*) after a 15 min continuous light illumination at an intensity of 2 J/cm^2^ as compared to RB treatment only. Remarkably, the nanohybrids at concentration 0.3 mg/mL completely eradicated a 21-day old biofilm after 30 min of irradiation at an intensity of 20 J/cm^2^ [52].

#### 2.3.4. Nanomaterials with Combination of Light and Environmental pH Stimulation

The combination therapy concept has attracted significant interest to increase treatment efficacy. A study by Hu et al. provided an example of the ability to attack *S. aureus* (MRSA) biofilms and kill the bacteria by using near infrared light (NIR) and environmental pH [74]. The authors created a coating consisting of zwitterionic gold nanoparticles that stayed as a dispersion of positively charged particles in normal pH, but turned into negatively charged particles at acidic pH (c.a. 5.5) in the bacterial biofilm. This conversion helped the particles to adhere electrostatically to the positively charged MRSA bacteria and to form aggregates. NIR irradiation then allowed the aggregates to convert and concentrate the photon energy into heat, causing local disruption of the biofilm. Meanwhile, the particles remained as well-dispersed particles in the normal pH and hence minimized photothermal damage to healthy tissue [74].

**Figure 7:**
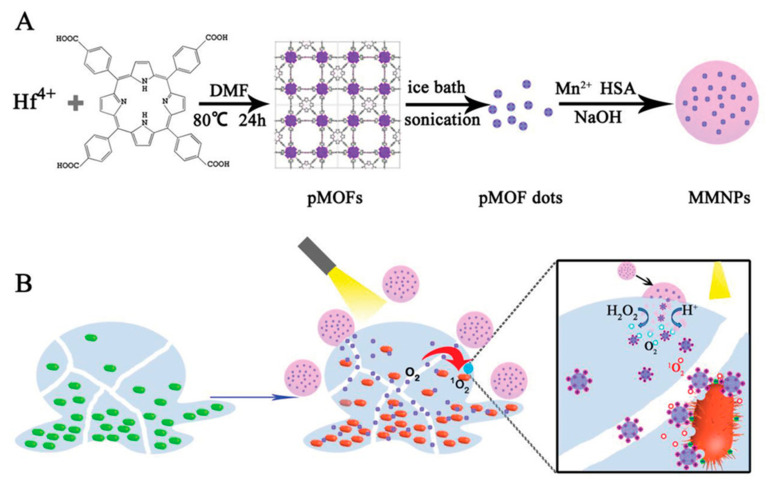
(**A**) Schematic illustration of nanomaterial fabrication for a multi-component nanoplatform (MMNPs) containing porphyrin-containing metal–organic framework (pMOF) dots. (**B**) Schematic of the mechanism of bacterial biofilm treatment by the nanoplatform (MMNPs) and external activation by NIR light. The MMNPs decomposed under an acidic environment, causing MnO_2_ degradation and pMOF dot release. The pMOF dots were then activated by NIR light to produce the reactive oxygen species. Additionally, in the presence of H_2_O_2_, MnO_2_ catalyzed H_2_O_2_ to form O_2_, enhancing the photodynamic therapy (reproduced from [75] with permission from John Wiley and Sons, 2019).

Deng et al. [75] also took advantage of the difference in pH between biofilm and healthy tissue to develop a sophisticated nanomaterial platform consisting of metal–organic framework (MOF) nanodots containing porphyrin and manganese dioxide embedded in human serum albumin for treating *E. coli* and *S. aureus* biofilms (Figure 7). Porphyrin absorbs light (maximum absorption occurs near a wavelength of 400 nm) and induces formation of singlet oxygen and other oxygen species in the presence of molecular oxygen [76,77]. MnO_2_ was designed to increase the O_2_ production via H_2_O_2_ catalyzation. Once the platform entered an acidic pH microenvironment in the biofilm, NIR irradiation would facilitate their decomposition, thereby releasing porphyrin-containing MOF dots (pMOF) while simultaneously generating oxygen. It was designed such that under the activation of NIR, the pMOF further oxidized the O_2_ to create ROS such as singlet oxygen ^1^O_2_, OH^-^ radicals, and O^2−^ radicals to eradicate biofilm. This platform was shown to decrease 82% and 84% of *E. coli* and *S. aureus* growth, respectively, after 15 min irradiation in the absence of H_2_O_2_. In the present of H_2_O_2_, 99% and 90% of *E. coli* and *S. aureus* growth was inhibited, respectively. The nanomaterials also disrupted 80% of 48 h old *S. aureus* biofilm using 15 min irradiation and H_2_O_2_. The material was further demonstrated to heal biofilm-infected abscesses in mice with no detectable damage to the surrounding healthy tissue [75].

## 3. Conclusions and Future Perspectives

As the recurrence of bacterial infection in revision surgeries remains clinically significant, new methods for treating device-associated biofilms are urgently needed. A large number of nanomaterials have emerged as promising materials in preventing biofilm formation, yet not all have been demonstrated to be able to destroy biofilms. This is mainly due to the fact that bacterial biofilms present a highly resistant community of bacteria embedded within a matrix of their self-secreted extracellular polymer substance (EPS). Treating these biofilms involves destroying their EPS and killing these bacteria and, thus, is significantly more challenging than preventing biofilm formation. Some nanomaterials have been shown to possess intrinsic biofilm-eradicating properties, such as those reviewed in this paper, yet combinations of intrinsic bactericidal or EPS-lysing properties with stimulation (such as local pH or magnetic fields or light) are expected to significantly increase the treatment efficacy. We have summarized the studies cited in this review in Table 1 to provide a quick reference to the key materials for this development of new nanomaterials.

Key issues in using nanomaterials for biofilm treatment are their cytotoxicity and the fact that their fates in the body remains incompletely understood. For example, it is known that cationic nanoparticles are often also toxic to mammalian cells and easily recognized by the reticuloendothelial system [78,79,80]. Nanomaterials’ toxicity strongly depends on their physicochemical properties such as shape, size, surface chemistry, structure, and agglomeration state [81]. Smaller sizes were often found with a higher level of toxicity, and agglomeration reduces the toxicity level [82]. Cytotoxicity also depends on the cell types in contact with the nanomaterials. For example, AuNP exhibit no significant toxic effects to dendritic cells, although they damage many other cell types [83]. The concerns of nanomaterial’s cytotoxicity and complex tissue distribution following administration are common for all nanomaterials and have been extensively reviewed elsewhere and therefore will not be reviewed here again; rather, we would like to draw the attention to an issue that has been largely overlooked in current biofilm treatment approaches, as below.

**Figure 8:**
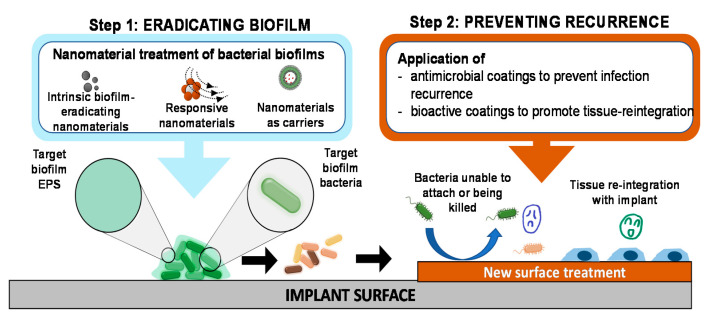
‘Eradicating-and-preventing’ approach to medical device-associated biofilms. After the biofilm has been eradicated (step 1), the surface of the implant should be further treated (step 2, for example by coating with antimicrobial agents or bioactive coating or with bioactive agents to enable rapid tissue-implant reintegration) to prevent infection recurrence. This proposed approach is essentially a combination of treating biofilms and preventing biofilm formation.

Nanomaterial research in treating bacterial biofilms is expected to continue developing more sophisticated or more complex mechanisms of destroying the EPS and killing the embedded bacteria. Considering the significantly higher rates of infection to revision surgery of infected implants compared to primary implants, it is important that future development should not overlook the prevention of recurrence after biofilm treatment. In our opinion, as shown in Figure 8, this prevention can be achieved through in situ functionalizing the treated surface with agents that accelerate tissue growth on the surface, based on the concept of ‘race for the surface’ [84,85].

Yet another challenge remaining is the fact that the use of chemical, thermal or photo-chemical reactions that allow coating process needs to be implemented directly on the implanted devices surrounded by host tissue. There are also some ‘mild’ coating methods [86,87,88,89,90,91,92,93] such as using catechol self-polymerization and surface functionalization chemistry, and photo-initiated crosslinking or surface-polymerization which should be further investigated to demonstrate biofilm treatment, recurrence prevention and tissue reintegration efficacy in vivo.

## Figures and Tables

**Table 1:** A summary of nanomaterials for treating biofilms on medical devices.

Activation Mechanism	Materials	Size and Shape	Preformed Bacterial Biofilm	Treatment Duration	Antibiofilm Efficacy		Ref.
					EPS Disruption	Bacterial Killing	
Intrinsic	Biosynthesized AgNPs from *P. zeylanica*	spheres 15–295 nm	24 h-*A. baumannii, E. coli,* and *S. aureus* 24 h-mixed species biofilm (*A. baumannii–S. aureus*)	24 h	88%, 67%, 78%, 64%	96% to 99%	[38]
	biosynthesized AgNPs from Acinetobacter calcoaceticus	spheres 4–40 nm	24 h-20 different pathogen bacterial biofilm	24 h	Up to 98%	NR	[39]
	AuNPs + proteinase K	spheres 11–27 nm	72 h-*Pseudomonas fluorescens* biofilm	24 h	74%	50%	[40]
	Chitosan NPs + O_3_ oil	NR	24 h-mixed-species biofilm (*S. mutans, E. faecalis,* and *C. albicans*)	8 days	NR	62.5%	[44]
	Chitosan NPs	spheres 220 ± 3 nm	7-day-*E. faecalis* biofilm	72 h	44%	75–80%	[45]
	PLGA + Octadecylamide NPs	spheres 217.7 nm	24 h-*S. mutants* biofilm	72 h	NR	Up to 73%	[46]
	α-TP + Phosphate α-TP + Trizma	700 nm	18 h-multispecies oral biofilms	2 min	NR	~45%	[47]
Nanocarriers	chitosan + DNase I + oxacillin	Spheres 166.7 nm	24 h-*S. aureus* biofilm	24 h	Up to 100%		[53]
	Chitosan NPs + ciprofloxacin (Cip)+ Fucoidan	spheres 235 ± 25 nm	48 h-*S. Paratyphi A* biofilm	48 h	62% in vitro 95% in vivo		[54]
	Gentamicin + phosphatidylcholine + AuNPs	Spheres ~180 nm	24 h-*P. aeruginosa* biofilm 24 h-*S. aureus* biofilm 24 h-*E. coli* biofilm 24 h-*L. monocytogenes* biofilm	24 h	68.75% 66.67% 69.23% 65.38%		[55]
	PLGA -Poly(lysin) + DNase I + Cip	spheres 251.9 nm	48 h-*P. aeruginosa* biofilm 24 h-*P. aeruginosa* biofilm	24 h	NR	43% 95%	[56]
	Silica + cinnamaldehyde NPs-peppermint oil capsule	NPs: ~150 nm Capsules: 6.7 ± 1.9 μm	24 h-*E. coli DH5, P. aeruginosa, S. aureus,* and *E. cloacae*	3 h	NR	90–100%	[57]
	copolymer Poly(oxanorborneneimide) bearing guanidine, amine, and tetraethylene glycol monomethyl ether units + poly(maleic anhydride-alt-octadecene + carvacrol oil	Spheres: ~250 nm	24 h-*E. coli DH5, P. aeruginosa, S. aureus,* and *E. cloacae*	3 h	NR	90–95%	[58]
	Silica NPs + nitric oxide (NO)	Spheres 14–150 nm	48 h-*S. aureus* and *P. aeruginosa* biofilm	24 h	NR	75%	[59]
	polyethylene glycol + glucose + chitosan + sodium nitrite + NO	spheres 10 nm	24 h-*MRSA* biofilm	24 h	NR	75%	[60]
Magnetically responsive	iron oxide (IONs) + Zn IONs + Ag IONs + Fe	19.67 ± 0.72 nm 193.72 ± 6.15 nm 23.32 ± 1.17 nm	24 h-*S. aureus* biofilm	24 h	85%, 40%, 50%		[64]
	IONs + amine IONs + carboxylate IONs + isocyanate	Spheres 14–19 nm	24 h-old *S. aureus* biofilm	24 h	NR	28.1%, 33.5% 31.1%	[65]
	silver + iron oxide + magnetic	Spheres 45–55 nm	24 h-*E. coli* biofilm 24 h-*P. aeruginosa* biofilm		88% 90%		[66]
Local pH or exogenous H_2_O_2_	Fe_3_O_4_ + H_2_O_2_	spheres 500 nm	Overnight *P. aeruginosa* biofilm	2 h	82%		[67]
	IONs + H_2_O_2_	Spheres 213 ± 26 nm	19 h-*S. mutants* in vitro	5 or 10 min, twice daily, 43 h	57–60%	99.9%	[70]
	DMAEMA + BMA + PAA + Farnesol	Sphere ∼60 nm	24 h-*S. mutants* biofilms	44 h	50%	80%	[8]
Light/heat	Nanobubbles produced by AuNPs + tobramycin	70 nm	24 h-*B. multivorans* biofilm 24 h-*P. aeruginosa* biofilm 24 h-*S. aureus* biofilm	3 min laser and 24 h tobramycin	NR	98% 95% 96%	[71]
	phospholipid 1,2-dioleoyl-sn-glycero-3-phosphocholine+ phospholipid 1,2-dipalmitoyl-sn-glycero-3-phospho-(1′-rac-glycerol) AuNPs + tobramycin graphene quantum dot + AuNPs + tobramycin	182 ± 102 nm 39 ± 14 nm	24 h-*P. aeruginosa* biofilm	3 min laser and 24 h tobramycin	75%		[72]
				15 min laser exposure and 24 h tobramycin	62%		
	Ag/g-C_3_N_4_	g-C_3_N_4_ Nanosheet AgNPs: 6.5 nm	48 h-*S. aureus* biofilm	3 h	70%	100%	[73]
	Chitosan NPs + Rose Bengal	spheres 60 ± 20 nm	21-day-biofilm	15 min irradiation from Lumacare lamp at 540 ± 15 nm	40–60%		[52]
Light and environmental pH	Citrate-caped AuNPs	spheres 14 nm	24 h-*MRSA* biofilm in vitro 24 h-subcutaneous abscess	60 min 10 min NIR light irradiation for 7 days	92% 88%		[74]
	pMOF nanodots + porphyrin + MnO_2_ + human serum albumin + H_2_O_2_	Spheres 105 nm	48 h-*S. aureus* biofilm 2-day-subcutaneous abscess	15 min visible light irradiation	87.5% 99.9%		[75]

NR—not reported.
